# Strabismus-mediated primary archenteron invagination is uncoupled from Wnt/β-catenin-dependent endoderm cell fate specification in *Nematostella vectensis *(Anthozoa, Cnidaria): Implications for the evolution of gastrulation

**DOI:** 10.1186/2041-9139-2-2

**Published:** 2011-01-21

**Authors:** Shalika Kumburegama, Naveen Wijesena, Ronghui Xu, Athula H Wikramanayake

**Affiliations:** 1 Department of Biology, The University of Miami, 1301 Memorial Drive, Coral Gables, FL 33146, USA; 2 Department of Zoology, 2538 McCarthy Mall, The University of Hawaii at Manoa, Honolulu, HI 96822, USA; 3 These authors contributed equally to the paper

## Abstract

**Background:**

Gastrulation is a uniquely metazoan character, and its genesis was arguably the key step that enabled the remarkable diversification within this clade. The process of gastrulation involves two tightly coupled events during embryogenesis of most metazoans. Morphogenesis produces a distinct internal epithelial layer in the embryo, and this epithelium becomes segregated as an endoderm/endomesodermal germ layer through the activation of a specific gene regulatory program. The developmental mechanisms that induced archenteron formation and led to the segregation of germ layers during metazoan evolution are unknown. But an increased understanding of development in early diverging taxa at the base of the metazoan tree may provide insights into the origins of these developmental mechanisms.

**Results:**

In the anthozoan cnidarian *Nematostella vectensis*, initial archenteron formation begins with bottle cell-induced buckling of the blastula epithelium at the animal pole. Here, we show that bottle cell formation and initial gut invagination in *Nematostella *requires NvStrabismus (NvStbm), a maternally-expressed core component of the Wnt/Planar Cell Polarity (PCP) pathway. The NvStbm protein is localized to the animal pole of the zygote, remains asymmetrically expressed through the cleavage stages, and becomes restricted to the apical side of invaginating bottle cells at the blastopore. Antisense morpholino-mediated NvStbm-knockdown blocks bottle cell formation and initial archenteron invagination, but it has no effect on Wnt/ß-catenin signaling-mediated endoderm cell fate specification. Conversely, selectively blocking Wnt/ß-catenin signaling inhibits endoderm cell fate specification but does not affect bottle cell formation and initial archenteron invagination.

**Conclusions:**

Our results demonstrate that Wnt/PCP-mediated initial archenteron invagination can be uncoupled from Wnt/ß-catenin-mediated endoderm cell fate specification in *Nematostella*, and provides evidence that these two processes could have evolved independently during metazoan evolution. We propose a two-step model for the evolution of an archenteron and the evolution of endodermal germ layer segregation. Asymmetric accumulation and activation of Wnt/PCP components at the animal pole of the last common ancestor to the eumetazoa may have induced the cell shape changes that led to the initial formation of an archenteron. Activation of Wnt/ß-catenin signaling at the animal pole may have led to the activation of a gene regulatory network that specified an endodermal cell fate in the archenteron.

## Background

The origin of metazoans from a choanoflagellate-like protist and the vast diversification of this clade is a remarkable evolutionary chronology in the history of life on Earth. From relatively simple origins, metazoans have radiated to produce organisms with levels of physiological and morphological complexity unmatched in other multicellular forms that have emerged in several clades [1,2]. The unique character that enabled the evolution of complex metazoans is generally believed to be the process of gastrulation [3]. The evolution of gastrulation produced a functionally distinct internal cell layer, and the interaction between the different tissue layers most likely led to the induction of new cell types, tissues, and organs [3]. Beginning with the seminal observations of Ernst Haeckel, a number of models have been proposed to reconstruct the evolution of gastrulation [reviewed in 4]. Many of these models posit that activation of morphogenesis on one side of a hypothetical blastula-like "urmetazoan" enabled cells on the outside to internalize and form an archenteron [4]. Regardless of the details of the individual models, it is likely that a crucial step in the evolution of gastrulation was the co-option of a localized molecular asymmetry that was present in ancient embryos to effect the cell shape changes that led to cell ingression and/or epithelial bending. However, the nature of the primordial anisotropy that triggered initial gastrulation movements is not known, and no existing model provides a molecular explanation for the initial evolution of a functional gut [3,5].

One ancient polarity that is present in most metazoan eggs is the animal-vegetal (AV) or primary axis of the egg [3,6]. The animal pole is defined by the site of polar body release during meiosis, and the AV axis is polarized by asymmetric distribution of maternal factors in the form of RNA, protein or organelles that can impart differential developmental potentials to blastomeres derived from the different poles of the egg [3,6,7]. In most animals, the AV axis predicts the axial properties of the embryo and adult. For example, in bilaterians, a clade that includes most animal phyla, patterns of gastrulation morphogenesis vary but a majority of these organisms develop their endomesoderm from vegetal pole-derived blastomeres [3,7]. In Porifera and Placozoa, two animal phyla generally believed to be two of the earliest diverging clades [8,9], little is known about the relationship between initial egg polarity and larval/adult patterning [10,11]. Additionally, these animals lack a functional endodermal germ layer. The Cnidaria and Ctenophora are the two remaining extant basal, non-bilaterian metazoan phyla and recent phylogenomic analyses indicate that they diverged after the appearance of the Porifera and the Placozoa [8]. In these two taxa that are clear outgroups to the bilaterians [8], and the only non-bilaterians to undergo true gastrulation, endoderm specification and archenteron formation are initiated at the animal pole [3,7,12]. These observations have led to the proposal that formation of an archenteron and segregation of an endodermal germ layer evolved at the animal pole, and the mechanisms that regulate these processes were moved to the vegetal pole sometime after the divergence of the urbilaterian from the last shared common ancestor with cnidarians and ctenophores [7,13]. This idea is supported at the molecular level by the observation that the site of gastrulation in several bilaterians and cnidarians is marked by nuclear ß-catenin [3,13-18]. If archenteron formation and segregation of an endodermal germ layer evolved at the animal pole, then cnidarians and ctenophores are key taxa for gaining insights into the developmental mechanisms that led to the evolution of these processes.

The anthozoan cnidarian *Nematostella vectensis *has emerged as an important model organism for comparative molecular studies of embryonic development. Recent studies have identified several key cellular and molecular processes involved in initial germ layer segregation and archenteron formation in this animal [13,14,19-21]. Gastrulation is initiated in *Nematostella *by primary archenteron invagination at the animal pole of the embryo [20,21]. This primary invagination is induced by an apical constriction of cells at the future blastopore, leading to the formation of bottle cells and a subsequent buckling of the blastula wall [20,21]. It is well established that bottle cells play an important role in initiating gastrulation in embryos of diverse taxa, and components of the ß-catenin-independent Wnt/PCP pathway appear to play a particularly important role in regulating apical constriction of these cells during initial archenteron formation [22-26]. There are several "core" PCP genes that are conserved between vertebrates and *Drosophila *[27], and we have initiated studies to determine the roles of these genes in regulating early polarity events in *Nematostella*. Here, we focus on the core Wnt/PCP gene *NvStrabismus (NvStbm)*. We show that *NvStbm *is maternally expressed, and the NvStbm protein becomes localized to the animal pole of the zygote, and remains asymmetrically localized to the apical end of blastomeres at one side of the embryo during the early cleavage stages. At the initiation of primary invagination, NvStbm becomes restricted to the apical end of cells at the blastopore. Knockdown of NvStbm using antisense morpholinos blocks bottle cell formation and archenteron invagination but has no effect on cell fate specification. Strikingly, blocking the Wnt/ß-catenin pathway, which is required for endoderm specification in *Nematostella *[13,14] has no effect on primary archenteron invagination indicating that initial gastrulation morphogenesis can be uncoupled from endoderm cell fate specification in this embryo. We propose a novel model for the evolution of gastrulation in metazoans that is based on a deeply conserved organismic polarity, and evolutionarily conserved signal transduction pathways.

## Results

### Cloning and phylogenetic analysis of an *Stbm *ortholog from *Nematostella*

**Figure 1:**
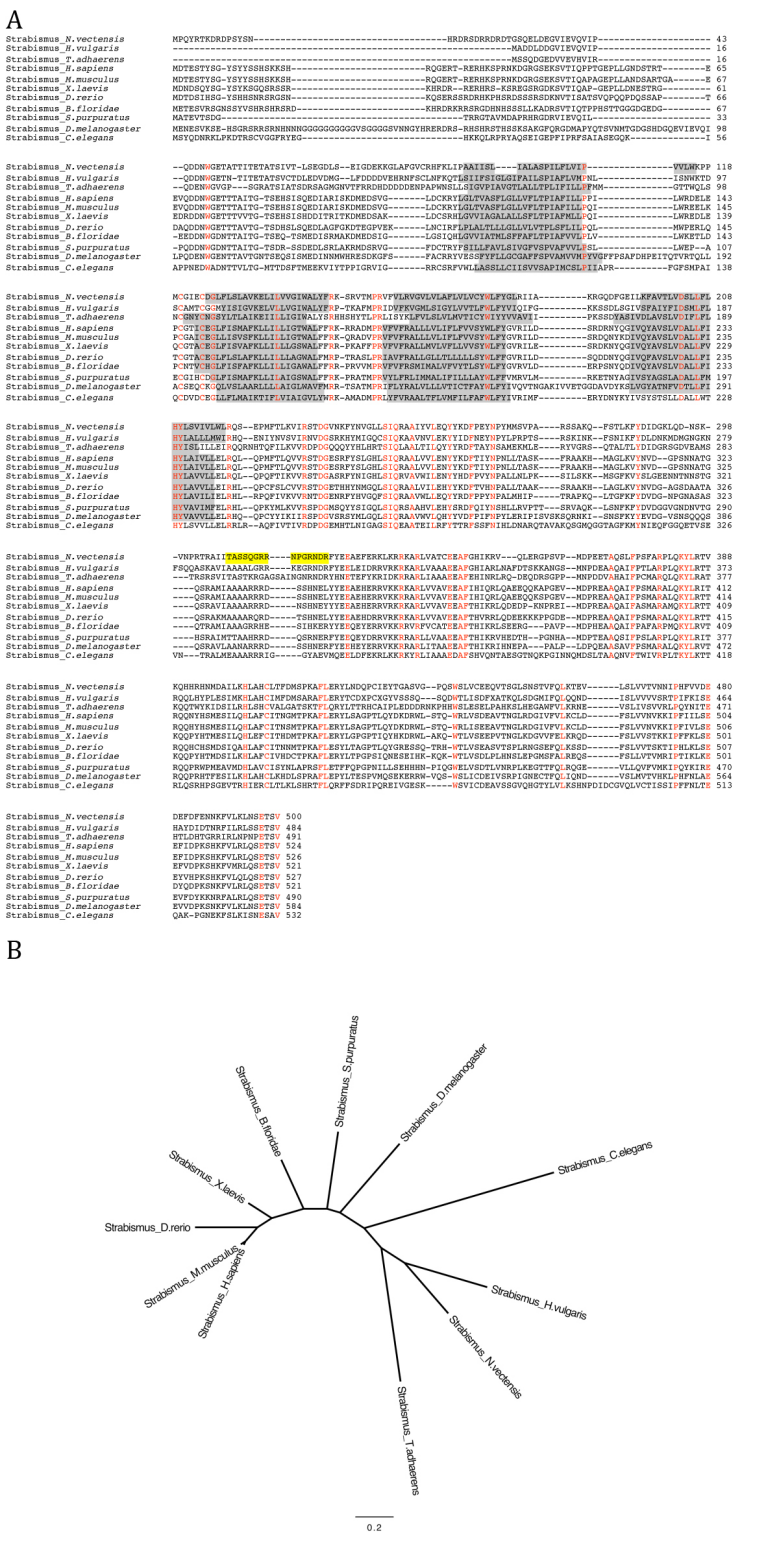
**Phylogenetic analysis of *Nematostella *Strabismus**. A) Alignment of NvStbm sequence with Stbm homologs from 10 metazoan taxa. The alignment was generated using ClustalW. Accession numbers used to obtain sequences for the alignment: Strabismus protein from *N. vectensis *(XP_001630420), *Homo sapiens *(NP_065068- 47% identity), *Mus musculus *(NP_808213- 48% identity), *Xenopus laevis *(NP_001083892- 47% identity) *Danio rerio *(NP_991313- 45% identity), *Branchiostoma floridae *(XP_002613474- 48% identity), *Caenorhabditis elegans *(NP_508500- 31% identity), *Drosophila melanogaster *(NP_477177- 41% identity), *Hydra vulgaris *XP_002160166 - 47% identity), *Trichoplax adhaerens *(XP_002107643 - 38% identity) and *Strongylocentrotus purpuratus *(41% identity). The percent identities of Stbm proteins from various taxa to NvStbm are indicated. Conserved amino acid residues across all nine taxa are shown in red. The regions highlighted in grey indicate the transmembrane regions and the region highlighted in yellow indicate the peptide sequence used to generate the anti NvStbm polyclonal antibody. B) Molecular phylogeny of Strabismus proteins from various metazoan taxa. Phylogenetic analysis of the NvStbm protein was carried out to determine orthology. Amino acid alignments including NvStbm and both vertebrate and invertebrate Stbm sequences were generated using MacClade [49]. A Bayesian Phylogenetic analysis was carried out using MrBayes 3.1 [50] with a run of one million generations, sampled every 100 generations. The summary consensus tree was generated in MrBayes using the last 7,500 trees and the posterior probabilities were calculated for this consensus tree. The posterior probability values for the consensus tree are shown.

Known bilaterian Stbm sequences were used to search an assembly of the *Nematostella *genome ([28] and Joint Genome Institute) and this resulted in the identification of several sequences coding for a putative Stbm. Predicted sequences were used to design PCR primers that were used to amplify a full length Stbm sequence from cDNA made from mRNA collected from mixed stage *Nematostella *embryos. Phylogenetic analysis confirmed that NvStbm is closely related to bilaterian, placozoan and cnidarian Stbm homologs, and that it is most likely a *Nematostella *ortholog of Strabismus (Figure 1A, B). NvStbm shows between 31 and 48% identity to various metazoan Stbm proteins and contains the four hydrophobic transmembrane domains and the highly conserved PDZ binding motif at the carboxy terminus (Figure 1A). Further searches of *Nematostella *genome sequences failed to reveal other related sequences. Using the available databases we were unable to identify homologs of this gene from sponges or ctenophores. The phylogenetic position of placozoans and ctenophores with respect to sponges, cnidarians and bilaterians is controversial and remains poorly resolved [8,9,29]. But our data indicate that a Stbm homolog was present in the last common ancestor to placozoans, cnidarians and bilaterians, and if the coelenterate clade is valid [8], then it is likely that ctenophores will also have a Stbm homolog, unless there has been a loss of Stbm in this taxon.

### Expression of NvStbm during *Nematostella *development

**Figure 2:**
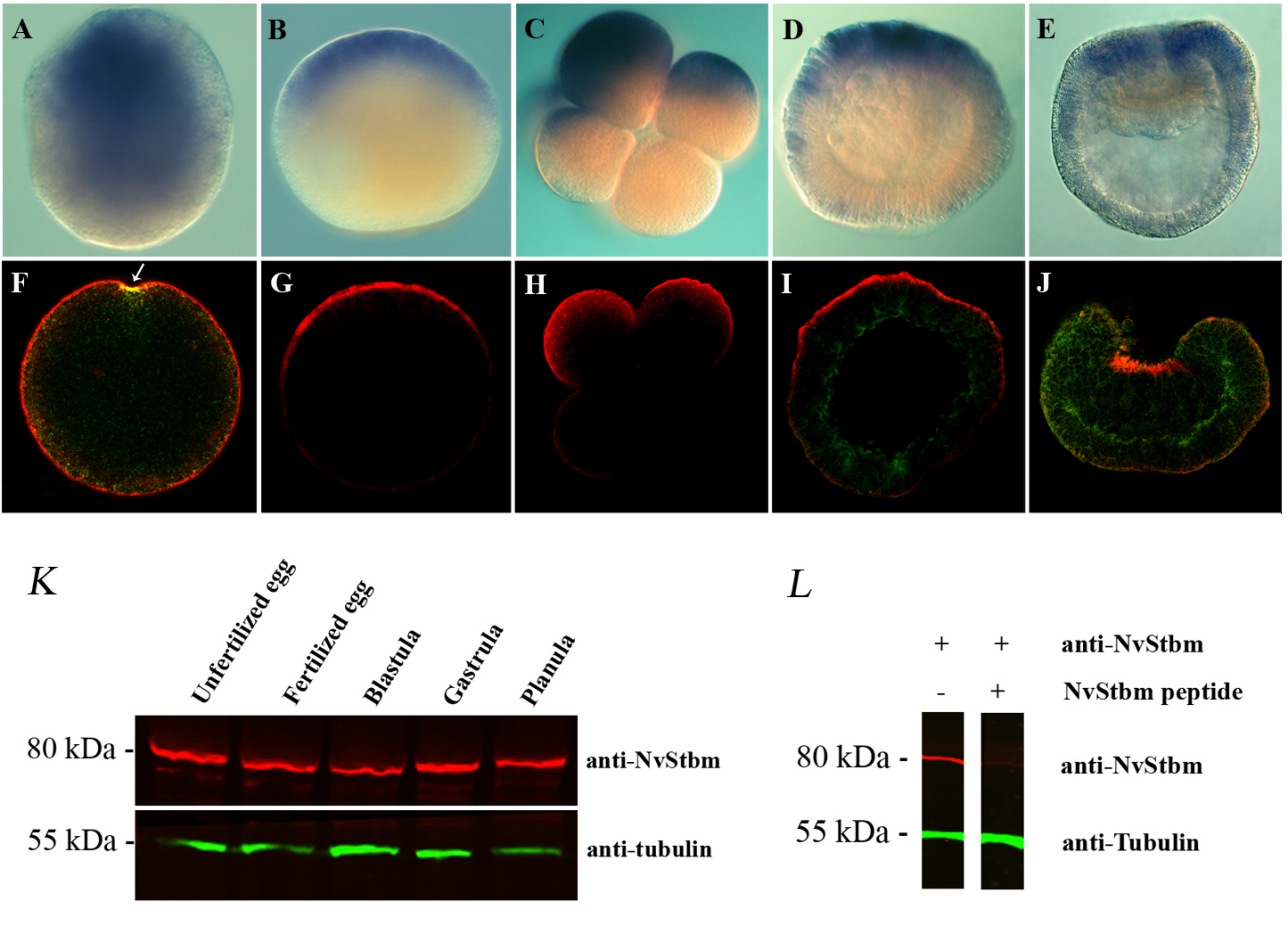
**Expression of NvStbm in *Nematostella *eggs and embryos**.(A-E) Whole mount RNA *in situ *hybridization for NvStbm. (A) Unfertilized egg. (B) Zygote. (C) 8-cell stage. (D) Blastula stage. (E) Gastrula stage. (F-J) NvStbm immunofluorescence in eggs and embryos (red). (F) Unfertilized egg. The female pronucleus at the animal pole (arrow) is labeled with anti-histone antibodies (green). (G) Zygote. (H) 8-cell. (I) Blastula. (J) Gastrula. The blastula and gastrula stages are also stained with phalloidin (green). At the gastrula stage the NvStbm is highly localized to the apical end of cells at the blastopore. The basal ends of these cells are deep inside the blastocoel at this time. (K) Western blot analysis of NvStvbm during early embryogenesis. The anti-NvStbm antiserum recognizes a single band at the expected size of 80 kD. The NvStbm protein is maternally expressed and is expressed at all the examined stages. Anti ß-tubulin was used as a loading control. (L) Preadsoption test of the specificity of the affinity purified anti NvStbm antibodies. Preadsorption of the affinity-purified anti-NvStbm polyclonal antibodies with ten fold molar excess of the NvStbm peptide used as the antigen results in elimination of staining of the 80 kD protein band observed in the control lane incubated with the anti-NvStbm antibodies. Anti ß-tubulin was used as a loading control.

Expression analysis of core PCP components in *Nematostella *using whole mount *in situ *hybridization (WMISH) revealed that the *Nematostella Stbm *ortholog is expressed in an intriguing pattern corresponding to the site of primary invagination and bottle cell formation (Figure 2). *NvStbm *mRNA is broadly expressed in unfertilized eggs with a consistent slight asymmetry towards one side (Figure 2A). After fertilization and during early embryogenesis *NvStbm *transcripts show a striking asymmetric expression pattern, and by the early gastrula stage (12 to 14 hours post fertilization, hpf) the transcripts accumulate around the blastopore, suggesting a role in gastrulation (Figure 2B-E). To examine the subcellular localization of the NvStbm protein, we generated affinity-purified anti-rabbit polyclonal antibodies to a peptide sequence corresponding to amino acids 308 to 322 of the NvStbm protein (Figure 1A). Western blots of *Nematostella *egg and embryo extracts showed that the antibodies recognized a band of 80 kD, which is the predicted size of the NvStbm protein (Figure 2K). The NvStbm protein is expressed maternally, and at relatively even levels during early embryogenesis and through the planula stage (Figure 2K). The specificity of the antibodies was demonstrated by preadsorption of the antiserum with the immunizing peptide, which resulted in the elimination of staining of the 80 kD band on Western blots (Figure 2L). Immunostaining of *Nematostella *eggs and early embryos using affinity-purified anti-NvStbm polyclonal antibodies revealed that NvStbm protein expression is congruent with transcript expression (Figure 2F-J). Double immunostaining for NvStbm and the female pronucleus in unfertilized eggs showed that NvStbm is enriched at the animal pole cell membrane (Figure 2F), and the protein remains asymmetrically localized at the apical cell surface of blastomeres on one side of the embryo (Figure 2G-I). At the early gastrula stage the NvStbm protein is clearly localized to the apical end of bottle cells at the blastopore (Figure 2J). NvStbm localization closely corresponded to the spatial expression of NvDishevelled (NvDsh)[13], a known partner of Stbm in Wnt/PCP signaling [27]. The lack of morphological landmarks during the early cleavage stages and at the blastula stage made it difficult to determine if NvStbm expression is strictly restricted to the animal pole blastomeres prior to gastrulation initiation. But the localization of NvStbm to the animal pole at the zygote stage and the early gastrula stage made it likely that the expression of the protein is restricted to the apical end of animal pole blastomeres. In sum, these results showed that NvStbm is maternally expressed and that the protein becomes localized to the apical end of the bottle cells at the blastopore, suggesting a role for NvStbm in the regulation of apical constriction of these cells.

### NvStbm knockdown blocks primary invagination

**Figure 3:**
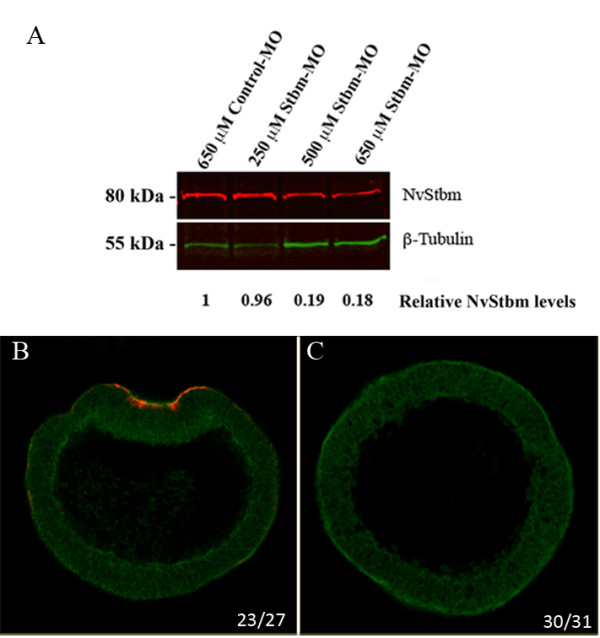
**Analysis of the efficacy of NvStbm knockdown using antisense NvStbm morpholinos**. (A) Western blot analysis was used to determine the efficacy of the NvStbm-MO. Individual protein band pixel intensities were measured using the Odyssey software (LI-COR, Lincoln, NE, USA). These intensities were used to calculate the ratios of NvStbm/ß-tubulin and normalized to determine the fold difference of each NvStbm band. This analysis showed that there is approximately a five-fold decrease of the endogenous Stbm protein in NvStbm-MO-injected (650 μM) embryos compared to embryos injected with the Control-MO (650 μM). (B, C) Scanning confocal microscopical analysis of Control- and NvStbm-MO injected embryos immunostained with affinity-purified NvStbm antibodies. (B) NvStbm antibody stained Control- MO-injected (650 μM) embryos showed strong NvStbm expression at the apical end of invaginating cells at the blastopore while (C) a majority of NvStbm-MO (650 μM) injected embryos did not show NvStbm expression. Also, NvStbm-MO injected embryos did not undergo initial archenteron invagination. The numbers in (B) represent the number of cases with NvStbm staining, and the numbers in (C) represent the number of cases without NvStbm staining.

**Figure 4:**
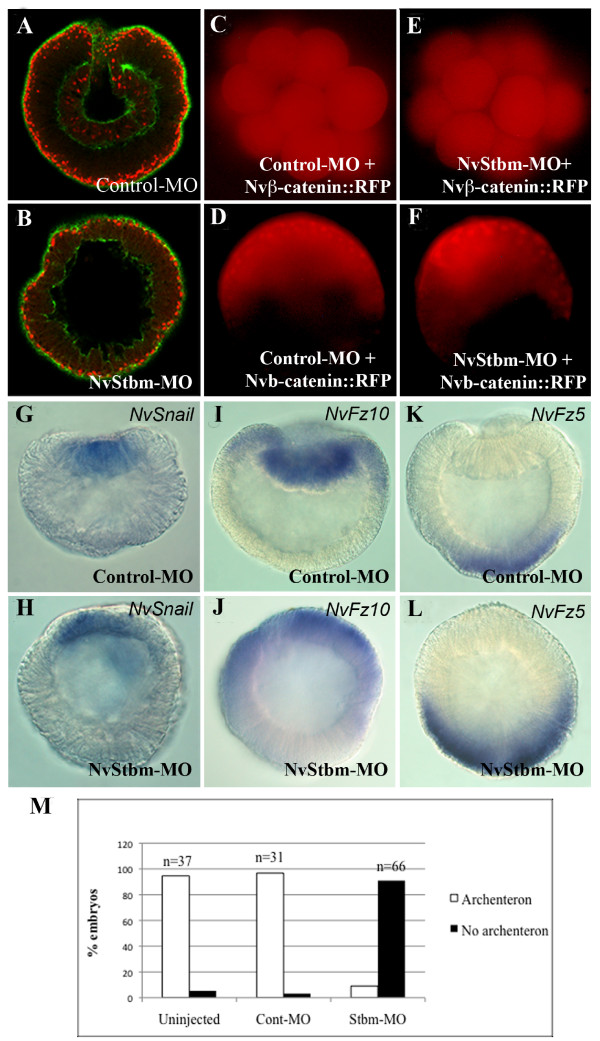
**The effect of NvStbm knockdown on endoderm specification and primary invagination in *Nematostella *embryos**. (A) Control-MO injected gastrula at 12 to 14 hpf. (B) NvStbm-MO injected embryo at 12-14 hpf. Embryos are stained with phalloidin (green) and propidium iodide (red). (C, D, E, F) Embryos co-injected with *Nvß-catenin::RFP *RNA and with either Cont-MO or NvStbm-MO. (C) In embryos injected with the Cont-MO, initially Nvß-catenin::RFP is expressed in all blastomeres as previously shown. (D) By the blastula stage Nvß-catenin::RFP is stabilized in animal-half derived blastomeres and enters the nuclei in these cells. Similar expression dynamics are seen in NvStbm-MO and *Nvß-catenin::RFP *RNA injected embryos at the early cleavage stage (E) and blastula stage (F), indicating that knockdown of NvStbm does not affect Wnt/ß-catenin signaling. (G-L) Analysis of gene expression in Control- and NvStbm-MO injected embryos. Similar to Cont-MO injected embryos that express *NvSnail *(G) and *NvFz10 *(I) in the endoderm, NvStbm-MO injected embryos also express *NvSnail *(H) and *NvFz10 *(J) but show no signs of archenteron invagination. *NvFz5 *expression in Cont-MO (K) and NvStbm-MO (L) injected embryos. (M) Quantification of the gastrulation phenotype in embryos using confocal microscopy shows that in contrast to Uninjected- (94.6%, n = 37) and Control-MO injected (96.8%, n = 31) embryos which gastrulated normally, very few NvStbm-MO injected embryos displayed an archenteron (9%, n = 66).

To test if NvStbm has a role in bottle cell formation and gastrulation, we used a translation-blocking NvStbm antisense morpholino (NvStbm-MO) to knockdown NvStbm protein levels. The efficacy of the NvStbm-MO in knocking down NvStbm protein expression was tested using Western blots and scanning confocal microscopy (Figure 3A-C). This analysis showed that by the blastula stage, injection of NvStbm-MO at 650 μM resulted in knockdown of NvStbm protein to approximately 20% of that of embryos injected with Control-MO (Figure 3A). We noted that on the Western blots, there were uneven levels of ß-tubulin that we used as an internal control for quantifying NvStbm knockdown following MO injections. The cause for this fluctuation was uncertain. Hence, to confirm the efficacy of NvStbm knockdown following NvStbm MO injection on a per-embryo basis, we used scanning confocal microscopy to carefully examine NvStbm immunostained Control- and NvStbm-MO-injected embryos at the early gastrula stage. This analysis showed that while Control-MO (650 μM) injected embryos showed strong NvStbm protein expression at the apical end of invaginating cells at the blastopore (Figure 3B), a majority of NvStbm-MO (650 μM) injected embryos did not show NvStbm protein expression (Figure 3C). We also noted that the NvStbm-MO injected embryos did not undergo initial archenteron invagination (Figure 3C). To further examine this phenotype, fertilized eggs injected with NvStbm-MO (650 μM) or Control-MO (650 μM) were collected at early (12 to 14 hpf) or mid gastrula (16 to 18 hpf) stages and analyzed using morphological and molecular criteria. Extensive analysis using scanning confocal microscopy showed that while Control-MO-injected embryos gastrulated normally (Figure 4A), most NvStbm morphants failed to gastrulate (Figure 4B), and did not form bottle cells. The blastula epithelium of NvStbm morphants remained columnar, and no cells or cell processes were seen extending into the blastocoel (Figure 4B). NvStbm-morphants displayed no signs of inward buckling of the blastula wall typical of primary invagination, and the apically located nuclei failed to migrate to the basal end of the cells as normally seen during bottle cell formation in *Nematostella *embryos (Figure 4B) [21]. This phenotype was highly reproducible and was evident in a majority of the NvStbm-MO-injected embryos (Figure 4M). These results demonstrated that reduced NvStbm protein levels disrupted bottle cell formation and completely blocked initial archenteron invagination.

### NvStbm knockdown does not affect ß-catenin nuclearization and endoderm specification

In bilaterians endoderm specification and morphogenesis are tightly coupled, and usually cell fate specification precedes morphogenesis [30-32]. Endoderm cell fate specification in *Nematostella *requires Wnt/ß-catenin signaling [13,14], and studies have shown that there is crosstalk between the Wnt/ß-catenin and the Wnt/PCP pathways [33]. This raised the possibility that NvStbm knockdown may affect Wnt/ß-catenin signaling and thereby block gastrulation by disrupting endoderm specification. To examine this we first analyzed embryos co-injected with *Nvß-catenin::RFP *mRNA (0.3 μg/μl) and NvStbm-MO or Control-MO to determine if NvStbm-knockdown affected the nuclearization of ß-catenin in blastomeres at the animal pole [13,14]. Analysis of these embryos showed that there was no difference in the Nvß-catenin::RFP expression pattern between NvStbm morphants, and control embryos (Figure 4C-F). Consistent with this observation, WMISH using several endoderm- and ectoderm-specific markers showed normal gene expression patterns in NvStbm morphants compared to controls (Figure 4G-L). These results indicated that failure of primary archenteron invagination in NvStbm morphant embryos was most likely not due to disruption of ß-catenin-dependent endoderm specification.

### *Nematostella *embryos undergo primary archenteron invagination in the absence of endoderm cell fate specification

**Figure 5:**
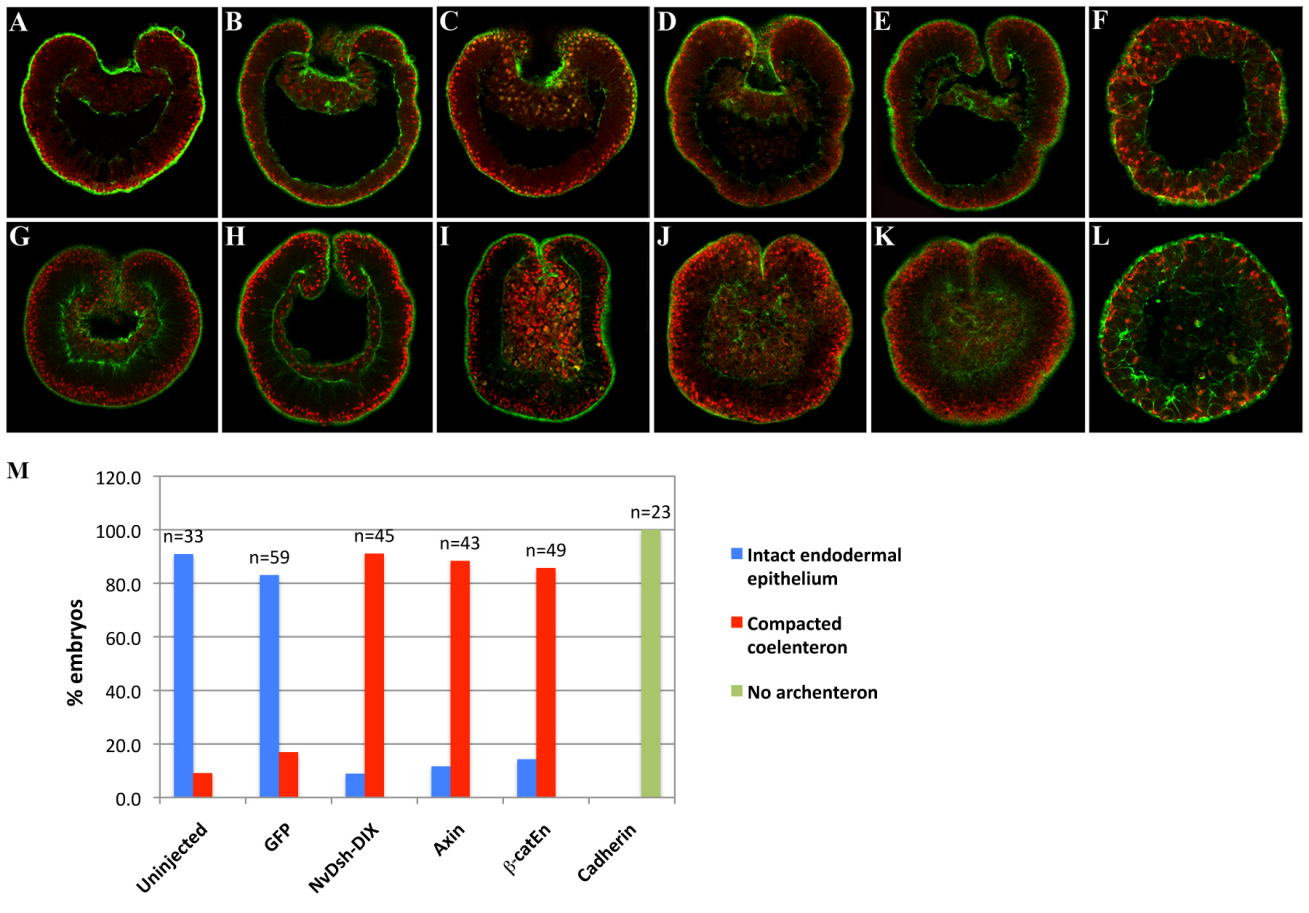
***Nematostella *embryos inhibited in ß-catenin signaling fail to specify endoderm, but undergo initial archenteron invagination**. (A-L) Morphological analysis of early (A-F) and late (G-L) gastrula stage embryos that are uninjected controls (A, G), or injected with *GFP *(B, H), *NvDsh-DIX::GFP *(C, I), *SpAxin *(D, J), *Xß-cat-Eng *(E,K), or *LvCadherin *(F, L) mRNA. Nuclei are stained with propidium iodide (red), and F-actin with phalloidin (green). Similar to controls (A, B), NvDsh-DIX (C), SpAxin (D) and Xß-cat-Eng (E) overexpressing embryos develop a primary invagination unlike LvCadherin (F) overexpressing embryos. However, while controls develop a normal endodermal epithelium at the late gastrula stage (G, H), NvDsh-DIX (I), SpAxin (J) and Xß-cat-Eng (K) overexpressing embryos have compacted coelenterons. Cadherin overexpressing embryos do not show any primary invagination and never develop an endodermal epithelium (L). (M) Quantification of the different phenotypes in mid and late gastrula stage embryos observed using confocal imaging clearly show that 91.1% (n = 45) of *NvDsh-DIX *RNA-injected embryos, 88.4% (n = 43) of *SpAxin *RNA-injected embryos and 85.7% (n = 49) of *ß-catEn *RNA-injected embryos cannot maintain the gut epithelium in contrast to 90.9% (n = 33) of uninjected and 83% (n = 59) of *GFP *RNA-injected embryos which show a normal endodermal epithelium. None of the *Lvcadherin *RNA injected embryos gastrulated (n = 23).

**Figure 6:**
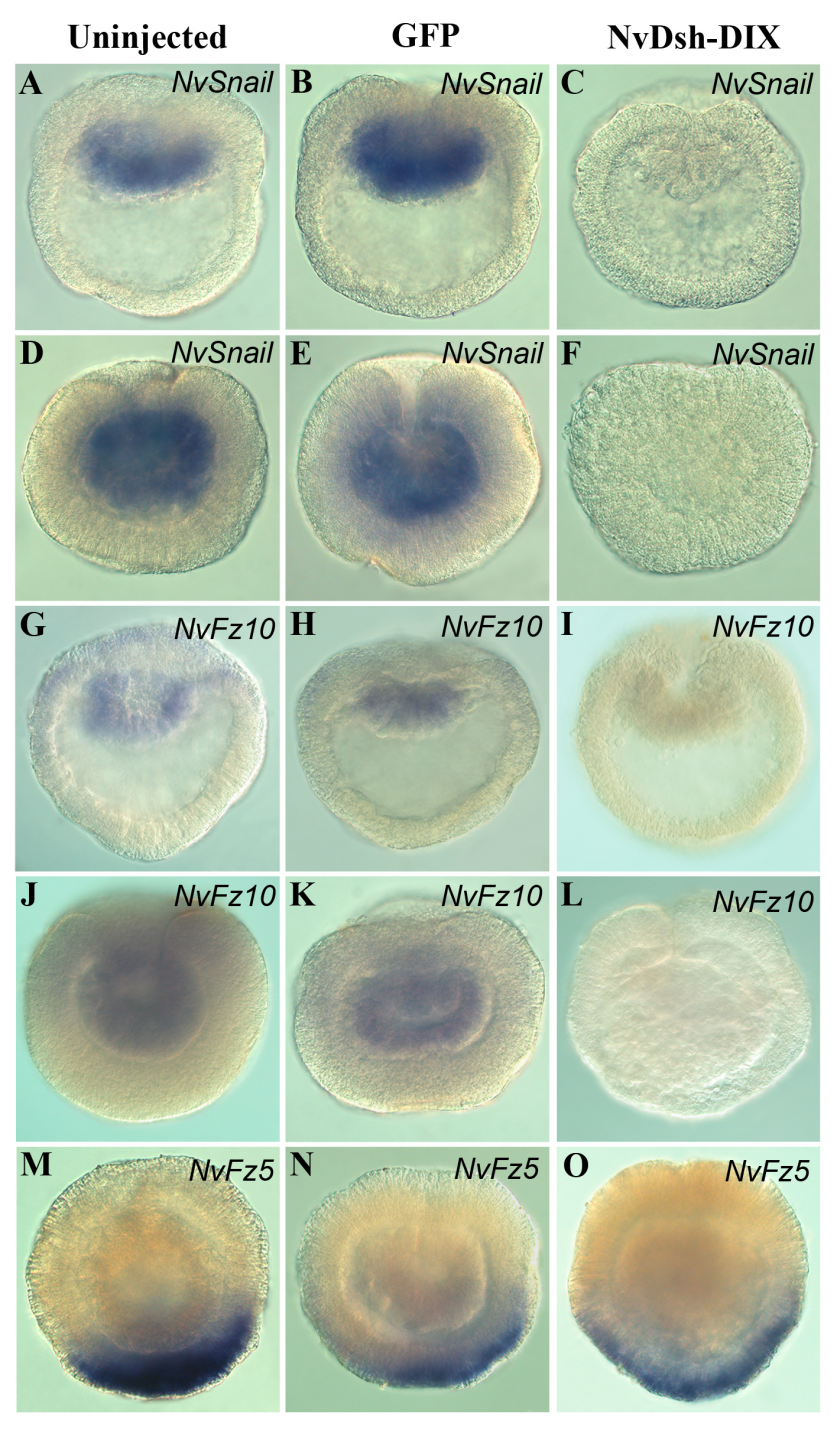
**Expression of endodermal and ectodermal genes in control and ß-catenin signaling-disrupted *Nematostella *embryos**. (A-O) WMISH for endodermal and ectodermal markers in uninjected, *GFP *mRNA- and *NvDsh-DIX::GFP *mRNA-injected embryos. Control uninjected (A, D, G, J) and *GFP *mRNA injected (B, E, H, K) embryos show expression of *NvSnail *and *NvFz10 *in the early A, B, G, H) and mid (D, E, J, K) gastrula stages. In contrast, *NvDsh-DIX::GFP *mRNA-injected embryos do not express these markers at the early (C, I) or mid (F, L) gastrula stages. Normal expression of the ectodermal marker *NvFz5 *is seen in uninjected (M), *GFP *mRNA injected (N), and *NvDsh-DIX::GFP *mRNA (O) injected embryos.

*NvStbm *is maternally expressed and hence its early expression would be independent of Wnt/ß-catenin signaling and endoderm specification. The maternal expression of NvStbm raised the possibility that if signaling by this protein through the Wnt/PCP pathway was independent of Wnt/ß-catenin signaling, then selectively blocking this branch of Wnt signaling would have no effect on bottle cell formation and initial archenteron invagination. To test this possibility we examined archenteron formation in embryos inhibited in Wnt/ß-catenin signaling. Previous studies blocking Wnt/ß-catenin signaling by overexpressing Cadherin or NvDsh-DIX, a Dsh dominant-negative in the Wnt/ß-catenin pathway, have produced somewhat different outcomes [13,14]. We have previously shown that *Nematostella *embryos overexpressing Cadherin did not express endodermal markers, and did not gastrulate [13,14]. Similarly, embryos overexpressing NvDsh-DIX did not express endodermal markers, but in contrast to Cadherin-overexpressing embryos, these embryos had a blastopore but lacked a visible archenteron [13]. The presence of a blastopore in NvDsh-DIX-overexpressing embryos was unexpected, and hence we used confocal microscopy to carefully analyze these embryos when control embryos were at early, mid, and late gastrula stages to determine the extent of archenteron formation in the absence of nuclear ß-catenin signaling. We observed that similar to uninjected and *GFP *RNA-injected control embryos, *NvDsh-DIX::GFP *RNA-injected embryos at the early gastrula stage were able to undergo normal initial archenteron invagination (Figure 5A-C). However, while control embryos possessed a distinct endodermal epithelium at mid to late gastrula (20 to 22 hpf) stages (Figure 5G, H, M), *NvDsh-DIX::GFP *RNA-injected late gastrula stage embryos lacked an endodermal epithelium, and displayed compacted coelentera (Figure 5I, M). We used WMISH to examine the extent of endoderm specification in *NvDsh-DIX *mRNA-injected embryos, and significantly, these embryos failed to express *NvSnail *and *NvFz10 *endoderm markers at early and mid gastrula stages compared to controls (Figure 6A-L). The lack of endodermal marker expression was most likely not from a delay in gene expression due to mRNA injections because *GFP *mRNA-injected embryos had normal endodermal gene expression (Figure 6B, E, H, K). Moreover, the *NvFz5 *ectodermal marker was expressed in *NvDsh-DIX *mRNA-injected embryos in a spatial expression pattern similar to controls (Figure 6M-O). Embryos injected with *SpAxin::GFP *and *Xß-catEn *mRNA, two proven methods to specifically downregulate Wnt/ß-catenin signaling [34,35] showed a phenotype similar to embryos overexpressing NvDsh-DIX (Figure 5D,E,J,K,M) supporting the idea that *Nematostella *embryos can undergo initial archenteron invagination in the absence of nuclear ß-catenin and endodermal cell fate specification. In normal embryos, after initial archenteron invagination the basal ends of the bottle cells at the edge of the blastopore develop processes that interact with the inside wall of the embryo to extend the archenteron toward the aboral pole of the embryo [21]. In embryos with disrupted Wnt/ß-catenin signaling, the archenteron does not extend most likely due to the absence of endodermal cell fate specification. Moreover, in these embryos the epithelium of the archenteron loses its integrity, indicating that while Wnt/ß-catenin signaling is not required for initial archenteron invagination, this signaling pathway is still needed for maintenance of the endodermal epithelium. We conclude that in *Nematostella*, bottle cell formation and primary archenteron invagination can occur in the absence of prior endoderm specification, but that Wnt/ß-catenin signaling is required for maintenance of the integrity of the endodermal epithelium.

We had previously reported that *LvCadherin *and *Xß-catEn *mRNA-injected embryos failed to gastrulate based on the lack of an archenteron [14]. In the experiments where we blocked nuclear ß-catenin by overexpressing cadherin, we consistently saw loss of endoderm gene expression and a failure of gastrulation in *Nematostella*, phenotypes we have reproduced in this study (Figure 5F, L). However, if primary archenteron invagination is uncoupled from endodermal cell fate specification, then LvCadherin-overexpressing embryos should produce bottle cells and consequently undergo primary invagination, but we have never observed this (Figure 5F, L, M). A possible explanation for this discrepancy comes from recent work that has shown that certain cadherins can affect the actin cytoskeleton [36]. Hence, it is possible that the lack of bottle cells and primary invagination in cadherin-overexpressing embryos is due to LvCadherin disrupting the cortical actin in bottle cells, thereby blocking apical constriction of these cells.

Our observations in the current study that Xß-catEn-overexpressing embryos consistently form a primary invagination contradict a result from our previous report [14]. In the previous study we used light microscopy to analyze the morphology of *Nematostella *embryos. These embryos are relatively opaque. Hence it is possible that the discrepancy between the results of the previous study and the current study is because when we previously examined Xß-catEn-overexpressing embryos the blastocoel appeared hollow because the invaginated archenteron had lost its epithelial integrity, and this unusual phenotype was undetectable using light microscopy. In the current study, examination of many *Xß-catEn *mRNA-injected embryos using scanning confocal microscopy clearly showed that these embryos formed a primary invagination, but these archentera lost their epithelial integrity by the late gastrula stage (Figure 5E, K). By using the Dsh-DIX dominant-negative, Axin and Xß-catEn to downregulate nuclear ß-catenin signaling, and through careful analysis of these phenotypes using scanning confocal microscopy and molecular markers, we show here that endoderm specification is not required for primary archenteron invagination in *Nematostella*, and moreover that the epithelium of this initial archenteron loses its integrity in the absence of nuclear ß-catenin.

## Discussion

In this study, we have presented results that implicate the core Wnt/PCP protein NvStbm in regulating primary archenteron invagination in *Nematostella*. We also show that in this cnidarian, NvStbm-regulated primary archenteron invagination is independent of Wnt/ß-catenin signaling-dependent endoderm cell fate specification, demonstrating an uncoupling of initial gut morphogenesis from endoderm cell fate specification. Our results support a model for the evolution of an archenteron in the last common ancestor to the eumetazoa where asymmetric localization and activation of Wnt/PCP signaling components at the animal pole induced the cell shape changes that led to epithelial buckling and morphogenesis of the first gut. Concomitant or subsequent activation of Wnt/ß-catenin signaling in animal pole blastomeres may have activated a gene regulatory network that specified endodermal cell fates in the archenteron.

### Strabismus and regulation of primary archenteron invagination in *Nematostella*

Cnidarians display more diverse gastrulation mechanisms than any other metazoan phylum [12]. However, a majority of examined anthozoan (and scyphozoan) species gastrulate by invagination, and it has been suggested that this mechanism was the primitive mode of gastrulation in cnidarians [12]. Typical of other anthozoans, archenteron formation in *Nematostella *is initiated by primary invagination at the animal pole of the embryo. Our results indicate that NvStbm plays a critical role in regulating primary invagination, and moreover, that this protein regulates this process by mediating the formation of bottle cells. NvStbm is expressed maternally and the protein becomes highly restricted to the apical end of bottle cells at the blastopore in *Nematostella *embryos. Knockdown of NvStbm protein levels results in the absence of apical constriction of cells at the animal pole, failure of bottle cell formation, and inhibition of primary archenteron invagination. To the best of our knowledge, Stbm has not been directly implicated in bottle cell formation in other metazoans, but this protein plays a critical role in regulating cell polarity and cell shape changes in many other cellular contexts [37-40]. Of relevance to the current study is that Stbm has been implicated in regulating the cell polarity mediating convergence and extension (CE) movements in vertebrates [37,38,40]. The mechanism of cell polarity regulation by Stbm during CE is not well understood, but it is believed to involve proteins in the Wnt/PCP pathway including Dsh, Prickle, RhoA, ROCK and Rac [40]. In many developmental contexts, apical constriction of bottle cells is regulated by RhoA-mediated activation of myosin II at the apical cortex [25]. We currently do not know how NvStbm regulates apical constriction in *Nematostella *through the Wnt/PCP pathway, but some observations suggest a role for NvDsh in this process. Dsh has been shown to regulate bottle cell formation in *Xenopus *embryos, and it is one of a relatively small number of proteins known to directly interact with Stbm [39,40]. NvDsh shows an expression pattern that closely matches the expression of NvStbm during early embryogenesis [13]. Moreover, blocking Wnt/PCP signaling in *Nematostella *by overexpressing Dsh-DEP, a Dsh dominant-negative in the Wnt/PCP pathway, also leads to failure of bottle cell formation and inhibition of initial archenteron invagination without affecting the Wnt/ß-catenin pathway [41]. Collectively these results suggest that an NvStbm/Dsh pathway may mediate myosin activation and regulate apical constriction and primary archenteron invagination in *Nematostella*. However, these possibilities and the role for other Wnt/PCP components in early gastrulation events in this cnidarian need to be addressed in future studies.

### Uncoupling of primary archenteron invagination and endoderm specification in *Nematostella*

**Figure 7:**
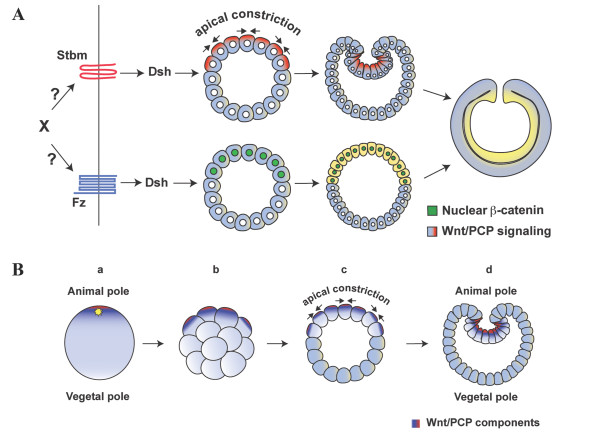
**Wnt signaling and the evolution of a functional gut**. (A) In *Nematostella*, initial archenteron invagination is regulated by bottle cell induction by NvStbm, and this can occur independently of endoderm cell fate specification mediated by Wnt/ß-catenin signaling. Both Wnt pathways are required for completion of gastrulation. It is possible that a single upstream ligand (X) or receptor (Fz) coordinates the activation of both branches of Wnt signaling during embryogenesis. (B) Model indicating the co-option of a polarity found in the oocytes of the last common ancestor to the eumetazoa to locally activate Wnt signaling at the animal pole. The centrosomes associated with the oocyte nucleus at meiosis served as a scaffold to localize critical Wnt pathway components to the apical pole (a). These components would be inherited by blastomeres at the animal pole (b), and their activation would drive apical constriction of these cells to form bottle cells (c), leading to the initial invagination of an archenteron (d). Endoderm specification mediated by Wnt/ß-catenin could have been coordinated with primary archenteron invagination by a single upstream ligand (A). Alternatively, endoderm specification could have occurred after the evolution of the primary invagination by the localized activation of the Wnt/ß-catenin pathway at the animal pole.

In bilaterian embryos, germ layer specification precedes gastrulation movements and usually these two events are very tightly linked [30-32]. Hence, the observation that endoderm cell fate specification and initial archenteron invagination can be uncoupled in *Nematostella *is striking and may provide insight into the evolutionary ontogeny of a functional gut in metazoans. We used three different reagents to inhibit the Wnt/ß-catenin pathway, but blocking endoderm cell fate specification through these perturbations had no effect on the initial invagination of the archenteron. The primary invagination in *Nematostella *is initiated by formation of bottle cells at the animal pole. Our results, therefore, strongly indicate that endoderm specification is not required for the formation of bottle cells and for initial archenteron formation by primary invagination. NvStbm is maternally expressed and becomes localized at the apical ends of the bottle cells at the blastopore. Since the expression of this protein would be independent of ß-catenin signaling and endoderm specification, we suggest that NvStbm can activate apical constriction of bottle cells and drive primary archenteron invagination without antecedent endoderm specification (Figure 7A). The mechanism through which Stbm regulates downstream signaling is not fully understood in any system [40]. In *Nematostella *it is likely that the expression of effectors of apical constriction of bottle cells downstream of Stbm are also uncoupled from endoderm specification, and it will be of great interest to identify these components and determine how they interact with NvStbm to regulate the formation of bottle cells.

Our observations in *Nematostella *raise the possibility that during evolution of gastrulation, initial morphogenesis of the archenteron could have occurred without prior segregation of an endodermal germ layer. The data presented here suggest that a maternally expressed Wnt/PCP pathway component localized to the animal pole in *Nematostella *embryos regulates primary archenteron invagination, while Wnt/ß-catenin signaling mediates endoderm specification. An important question, however, is whether the mechanisms we observe in *Nematostella *are restricted to anthozoans, or if they are more generally seen in the Cnidaria. There is no other anthozoan model system where mechanisms of endoderm specification and gastrulation are understood at the molecular level. Similarly, to the best of our knowledge, functional molecular analysis of early embryonic development has not been done in cubozoans, or in scyphozoans. The fourth cnidarian class comprises the hydrozoans, and from this group *Hydra *has proved to be an important cnidarian model for studying axial patterning in the adult. However, due to technical difficulties of obtaining eggs and embryos, *Hydra *is not easily amenable to molecular manipulation during the embryonic stages [12]. Over the past several years, the hydrozoan *Clytia hemisphaerica *has emerged as an important model system for studying hydrozoan embryogenesis [42]. Elegant work done in *Clytia *has established the importance of Wnt/ß-catenin signaling in endoderm specification in this organism [15,43]. In this hydrozoan, oral/endoderm specification is regulated by CheWnt3, which is localized to the animal pole of the unfertilized egg. Blocking CheWnt3 using an antisense morpholino eliminated nuclearization of ß-catenin and the expression of endodermal markers. However, while these embryos were delayed in gastrulation, by the planula stage they had what appeared to be a complete endodermal epithelium [43]. Similar to what we observed in *Nematostella *[13, this study], in *Clytia*, the archentera of embryos lacking nuclear ß-catenin did not express any markers of endoderm differentiation [43]. Interestingly, when the translation of CheFz1, a Frizzled homolog localized to the animal pole of the unfertilized *Clytia *egg, was blocked using an antisense morpholino, these embryos did not nuclearize ß-catenin or express markers for endoderm, and moreover, they did not show any signs of gastrulation [15]. Based on the effect of CheFz1 knockdown on the polarity of the embryo, these authors suggested that this Frizzled receptor was involved in endoderm specification through Wnt/ß-catenin signaling, as well as signaling through a Wnt/PCP pathway. In sum, these results suggest that morphogenesis of the gut and cell fate specification are also uncoupled in *Clytia, *and in this case as well, the activities are regulated by the two Wnt pathways. Gastrulation in *Clytia *and most hydrozoans involves ingression rather than invagination, but this process also involves bottle cell formation by apical constriction of cells at the blastopore [12,43]. It would be interesting to determine if *Clytia *Stbm orthologs and other components of Wnt/PCP signaling affect the formation of bottle cells in this embryo as seen in *Nematostella*.

### Localization of Wnt/PCP components to the animal pole, and the evolution of gastrulation

Bottle cell formation is a common strategy that is used to initiate epithelial bending in metazoan embryos [25]. In particular, bottle cell formation via apical constriction is widely used to initiate archenteron invagination in diverse taxa [25]. This highly conserved mechanism for initiating gastrulation has led to the suggestion that localized induction of bottle cells could have led to the initial formation of an archenteron [31]. However, a molecular mechanism for how the coordinated and spatially localized apical constriction of a group of cells could have occurred, that is based on deeply conserved organismic underpinnings has heretofore been missing. Recent studies have implicated Wnt/PCP pathway components in bottle cell formation during gastrulation initiation in *C. elegans*, sea urchins and *Xenopus *[22-25]. In this study, our data implicate a core component of the Wnt/PCP pathway in the formation of bottle cells in embryos of a modern descendant of one of the earliest diverging metazoan phyla, indicating a possible ancient co-option of this pathway for mediating apical constriction at one end of the embryo. Other studies have shown that there is an enrichment of Wnt pathway components at the animal pole of cnidarian eggs [15,43] raising an intriguing possibility for the initial evolution of the archenteron. There is increasing evidence that Wnt pathway components accumulate at centrosomes and cilia found in many epithelial cell types [44-46]. The animal pole is defined by the site of polar body release [6,7], hence oocytes of all metazoans should have centrosomes localized at this pole during meiosis. Interestingly, oocytes of some invertebrate deuterostomes have a flagellum at the animal pole, and it has been shown that the AV axis in these animals corresponds to the apical-basal polarity of the germinal epithelium, suggesting that the AV axis may be homologous to the apical basal polarity of epithelial cells [47,48]. The prevalence of this character in other taxa is unknown, but it is likely that cilia/flagella and the centrosomes associated with this organelle are homologous to the flagellar apparatus in the unicellular protist ancestor of metazoans. Thus, we propose that the centrosomes and flagellum of the protist last common ancestor of metazoans was the original polarity that was co-opted during metazoan evolution to enrich components of the Wnt pathway to one end of the eumetazoan oocyte (Figure 7B). Cell autonomous activation of Wnt/PCP signaling may have led to apical constriction-mediated cell shape change and initial archenteron invagination. Nuclearization of ß-catenin at the animal pole by these localized Wnt pathway components may have activated the expression of gene products that led to the segregation of a novel germ layer, and the evolution of a functional gut.

## Materials and methods

### Spawning and gamete handling

Spawning, gamete preparation and embryo culturing was done as previously described [13].

### DNA constructs

PCR was used to amplify full-length NvStbm and partial fragments of NvFz10 and NvFz5 from cDNA prepared from mixed-stage *Nematostella *embryo RNA (primer sequences and accession numbers are listed in Table 1). *SpAxin::GFP *was constructed by PCR-amplifying an *Axin *fragment from cDNA prepared from blastula stage *Strongylocentrotus purpuratus *embryos, and subcloning this fragment into pCS2 + GFP. *Nvß-catenin::RFP *was made by replacing the GFP sequence in Nvß-catenin::GFP [13] with RFP. NvDsh-DIX, Lvcadherin and Xß-catEn (Xenopus ß-catenin fused to the *Drosophila Engrailed *repressor domain) constructs have been described previously [13,18,35]. Constructs used for WMISH were cloned into pGEM-T. Additional details of construct synthesis are available upon request.

**Table 1:** Primer sequences used to clone various cDNAs used in this study

Gene or Construct	**Accession No**:	Primers	Template	Vector
*NvStbm *(in situ hybridization probe)	1526895:4	Forward 5' CGACAAAGCGAACCAATGTTTACTCT 3' Reverse 5' CCGACATCATGTAAGGGTTATACTCA 3'	Mixed stage *N.vectensis *cDNA	pGEM

*NvStbm *(Full-length)	1526895:4	Forward 5'GCCGGAATTCATGCCGCAATATCGTACCAAAGAC 3' Reverse 5' GCCTAGGCCTGACGGAGGTCTCCGAATTTAACTTG 3'	Mixed stage *N.vectensis *cDNA	pCS2^+^GFP

*NvFz10*	XP_001630630	Forward 5' AGTACGATCAGTGCGTCCCCCAATG 3' Reverse 5' CCGCCACGACTTGAATGTTTTTGTAG 3'	Mixed stage *N.vectensis *cDNA	pGEM

*NvFz5*	XP_001634995	Forward 5' GCGAAGAGATCACCATTCCCATGTGC 3' Reverse 5' ACCCACCAAACAGAGGAAGCCATTC 3'	Mixed stage *N.vectensis *cDNA	pGEM

*SpAxin*	XP_781992	Forward 5' GCCGGAATTCATGAGTCTAGAAGTGTATAGGTTC 3' Reverse 5' GCCGAGGCCTGTGATCATCGACAGATTCCACCTG 3'	Blastula stage *S. purpuratus *cDNA	pCS2^+^GFP

### mRNA synthesis, morpholinos and microinjection

Synthesis of mRNA was done as previously described [13,14]. Anti-sense morpholino oligonucleotides for NvStbm (NvStbm-MO; 5' GCGCTAAACTTGTTACAATCACAGC 3') and standard control morpholinos (5' CCTCTTACCTCAGTTACAATTTATA 3') were synthesized at Gene Tools (Philomath, OR, USA) (Table 1). The NvStbm MO was a translation blocking morpholino designed to bind to the 5' UTR of the NvStbm mRNA immediately upstream of the translation start site. Morpholinos and synthetic RNA were diluted in 40% glycerol, and control RNAs were injected at the same molar concentrations as the RNAs coding for the experimental constructs. Injected embryos were cultured at 25°C in 1/3X seawater.

### Antibody production and Western blot analysis

Anti-NvStbm polyclonal rabbit antibodies were raised against a selected sequence corresponding to amino acids 308 to 322 of NvStbm (NH2-TASSQGRRNPGRNDR-COOH), and affinity-purified using the immunizing peptide (Bethyl Labs, Montgomery, TX, USA). Western blots were performed as previously described [13,14]. Anti-NvStbm and anti-ß-Tubulin (DSHB, Iowa City, IA, USA) antibodies were used at 1:750 and 1:1000 dilutions, respectively. Goat anti-Rabbit IRDye 680 (1:12,000) and Goat anti-Mouse IRDye 800 (1:12,000) were used as secondary antibodies (LI-COR, Lincoln, NE, USA). Stained Western blots were analyzed using an Odyssey Infrared Imager and the Odyssey software (LI-COR).

### Immunochemistry, whole mount RNA in situ hybridization and microscopy

Immunochemistry and WMISH was performed as previously described [13]. Anti-NvStbm and mouse anti-histone antibodies (Millipore, MA, USA) were used at 1:100 and 1:250 dilutions, respectively. Immunostained embryos were examined using a Leica TCS SP5 scanning confocal microscope.

## Abbreviations

AV: Animal-vegetal; CE: Convergence and extension; Dsh: Dishevelled; Fz10: Frizzled 10; Fz5: Frizzled 5; GFP: Green fluorescent protein; Hpf: Hours post fertilization; Lv: *Lytechinus variegatus; *MO: Morpholino; Nv: *Nematostella vectensis; *PCR: Polymerase chain reaction; RFP: Red fluorescent protein; Sp: *Strongylocentrotus purpuratus; *Stbm: Strabismus; WMISH: Whole mount *in situ *hybridization; Wnt/PCP pathway: Wnt/Planar Cell Polarity pathway; *Xß-catEn*: *Xenopus *ß-catenin engrailed;

## Competing interests

The authors declare that they have no conflict of interest.

## Authors' contributions

SK, NW and AHW designed the research. SK, NW and RX performed the research. SK, NW and AHW analyzed the data and wrote the paper.

## References

[B1] King N The unicellular ancestry of animal development Dev Cell 2004 7 313 325 10.1016/j.devcel.2004.08.010 15363407

[B2] Rokas A The origins of multicellularity and the history of the genetic toolkit for animal development Annual Rev Genet 2008 42 235 251 10.1146/annurev.genet.42.110807.091513 18983257

[B3] Martindale MQ The evolution of metazoan axial properties Nat Rev Genet 2005 6 917 927 10.1038/nrg1725 16341072

[B4] Willmer P Invertebrate Relationships. Patterns in Animal Evolution 1990 Cambridge: Cambridge University Press

[B5] Wolpert L Gastrulation and the evolution of development Development 1992 7 13 1299370

[B6] Goldstein B Freeman G Axis specification in animal development BioEssays 1997 19 105 116 10.1002/bies.950190205 9046240

[B7] Martindale MQ Hejnol A A developmental perspective: changes in the position of the blastopore during bilaterian evolution Dev Cell 2009 17 162 174 10.1016/j.devcel.2009.07.024 19686678

[B8] Philippe H Derelle R Lopez P Pick K Borchiellini C Boury-Esnault N Vacelet J Renard E Houliston E Quéinnec E Da Silva C Wincker P Le Guyader H Leys S Jackson DJ Schreiber F Erpenbeck D Morgenstern B Wörheide G Manuel M Phylogenomics revives traditional views on deep animal relationships Curr Biol 2009 19 706 712 10.1016/j.cub.2009.02.052 19345102

[B9] Srivastava M Begovic E Chapman J Putnam NH Hellsten U Kawashima T Kuo A Mitros T Salamov A Carpenter ML Signorovitch AY Moreno MA Kamm K Grimwood J Schmutz J Shapiro H Grigoriev IV Buss LW Schierwater B Dellaporta SL Rokhsar DS The Trichoplax genome and the nature of placozoans Nature 2008 454 955 960 10.1038/nature07191 18719581

[B10] Ereskovsky AV The Comparative Embryology of Sponges 2010 New York: Springer

[B11] Schierwater B My favorite animal, *Trichoplax adhaerens.* BioEssays 2005 27 1294 1302 10.1002/bies.20320 16299758

[B12] Byrum CA Martindale MQ Stern C Gastrulation in the Cnidaria and Ctenophora Gastrulation from cells to embryo 2004 Cold Spring Harbor: Cold Spring Harbor Laboratory Press 33 50

[B13] Lee P Kumburegama S Marlow HQ Martindale MQ Wikramanayake AH Asymmetric developmental potential along the animal-vegetal axis in the anthozoan cnidarian, *Nematostella vectensis*, is mediated by dishevelled Dev Biol 2007 310 169 186 10.1016/j.ydbio.2007.05.040 17716645

[B14] Wikramanayake AH Hong M Lee PN Pang K Byrum CA Bince JM Xu R Martindale MQ An ancient role for nuclear ß-catenin in the evolution of axial polarity and germ layer segregation Nature 2003 426 446 450 10.1038/nature02113 14647383

[B15] Momose T Houliston E Two oppositely localised frizzled RNAs as axis determinants in a cnidarian embryo PLoS Biol 2007 5 e70 10.1371/journal.pbio.0050070 17355179 PMC1820609

[B16] Henry JQ Perry KJ Wever J Seaver E Martindale MQ ß-catenin is required for the establishment of vegetal embryonic fates in the nemertean, *Cerebratulus lacteus* Dev Biol 2008 317 368 379 10.1016/j.ydbio.2008.02.042 18387602

[B17] Schneider S Steinbeisser H Warga RM Hausen P Beta-catenin translocation into nuclei demarcates the dorsalizing centers in frog and fish embryos Mech Dev 1996 57 191 198 10.1016/0925-4773(96)00546-1 8843396

[B18] Logan CY Miller JR Ferkowicz MJ McClay DR Nuclear beta-catenin is required to specify vegetal cell fates in the sea urchin embryo Development 1999 126 345 357 9847248 10.1242/dev.126.2.345

[B19] Martindale MQ Pang K Finnerty JR Investigating the origins of triploblasty: "Mesodermal" gene expression in a diploblastic animal, the sea anemone, *Nematostella vectensis *(Phylum Cnidaria; Class Anthozoa) Development 2004 131 2463 2474 10.1242/dev.01119 15128674

[B20] Kraus Y Technau U Gastrulation in the sea anemone *Nematostella vectensis *occurs by invagination and immigration: an ultrastructural study Dev Genes Evol 2006 216 119 132 10.1007/s00427-005-0038-3 16416137

[B21] Magie CR Daly M Martindale MQ Gastrulation in the cnidarian *Nematostella vectensis *occurs via invagination not ingression Dev Biol 2007 305 483 497 10.1016/j.ydbio.2007.02.044 17397821

[B22] Lee JY Marston DJ Walston T Hardin J Halberstadt A Goldstein B Wnt/Frizzled signaling controls *C. elegans *gastrulation by activating actomyosin contractility Curr Biol 2006 16 1986 1997 10.1016/j.cub.2006.08.090 17055977 PMC2989422

[B23] Croce J Duloquin L Lhomond G McClay DR Gache C Frizzled5/8 is required in secondary mesenchyme cells to initiate archenteron invagination during sea urchin development Development 2006 133 547 557 10.1242/dev.02218 16396908

[B24] Choi SC Sokol SY The involvement of lethal giant larvae and Wnt signaling in bottle cell formation in *Xenopus *embryos Dev Biol 2009 336 68 75 10.1016/j.ydbio.2009.09.033 19782678 PMC2801549

[B25] Sawyer JM Harrell JR Shemer G Sullivan-Brown J Roh-Johnson M Goldstein B Apical constriction: A cell shape change that can drive morphogenesis Dev Biol 2010 341 5 19 10.1016/j.ydbio.2009.09.009 19751720 PMC2875788

[B26] Sherrard K Robin F Lemaire P Munro E Sequential activation of apical and basolateral contractility drives ascidian endoderm invagination Curr Biol 2010 20 1499 1510 10.1016/j.cub.2010.06.075 20691592 PMC4088275

[B27] Simons M Mlodzik M Planar cell polarity signaling: from fly development to human disease Annu Rev Genet 2008 42 517 540 10.1146/annurev.genet.42.110807.091432 18710302 PMC2814158

[B28] Sullivan JC Ryan JF Watson JA Webb J Mullikin JC Rokhsar D Finnerty JR StellaBase: the *Nematostella vectensis *Genomics Database Nucleic Acids Res 2006 34 Database issue D495 499 10.1093/nar/gkj020 16381919 PMC1347383

[B29] Dunn CW Hejnol A Matus DQ Pang K Browne WE Smith SA Seaver E Rouse GW Obst M Edgecombe GD Sørensen MV Haddock SH Schmidt-Rhaesa A Okusu A Kristensen RM Wheeler WC Martindale MQ Giribet G Broad phylogenomic sampling improves resolution of the animal tree of life Nature 2008 452 745 749 10.1038/nature06614 18322464

[B30] Rodaway A Patient R Mesendoderm: An ancient germ layer? Cell 2001 105 169 172 10.1016/S0092-8674(01)00307-5 11336666

[B31] Leptin M Gastrulation movements: the logic and the nuts and bolts Dev Cell 2005 8 305 320 10.1016/j.devcel.2005.02.007 15737927

[B32] Heisenberg C Solnica-Krezel L Back and forth between cell fate specification and movement during vertebrate gastrulation Curr Opin Gen Dev 2008 18 311 316 10.1016/j.gde.2008.07.011 PMC2706661 18721878

[B33] van Amerongen R Nusse R Towards an integrated view of Wnt signaling in development Development 2009 136 3205 3214 10.1242/dev.033910 19736321

[B34] Sakanaka C Weiss JB Williams LT Bridging of beta-catenin and glycogen synthase kinase-3beta by axin and inhibition of beta-catenin-mediated transcription Proc Natl Acad Sci USA 1998 95 3020 3023 10.1073/pnas.95.6.3020 9501208 PMC19687

[B35] Montross WT Ji H McCrea PD A ß-catenin/engrailed chimera selectively suppresses Wnt signaling J Cell Sci 2000 113 1759 1770 10769207 10.1242/jcs.113.10.1759

[B36] Nandadasa S Tao Q Menon NR Heasman J Wylie C N- and E-cadherins in *Xenopus *are specifically required in the neural and non-neural ectoderm, respectively, for F-actin assembly and morphogenetic movements Development 2009 136 1327 1338 10.1242/dev.031203 19279134 PMC2687464

[B37] Goto T Davidson L Asashima M Keller R Planar cell polarity genes regulate polarized extracellular matrix deposition during frog gastrulation Curr Biol 2005 15 787 793 10.1016/j.cub.2005.03.040 15854914

[B38] Jessen JR Topczewski J Bingham S Sepich DS Marlow F Chandrasekhar A Solnica-Krezel L Zebrafish *trilobite *identifies new roles for *strabismus *in gastrulation and neuronal movements Nat Cell Biol 2002 4 610 615 12105418 10.1038/ncb828 PMC2219916

[B39] Babstock R Strutt H Strutt D Strabismus is asymmetrically localized and binds to Prickle and Dishevelled during *Drosophila *planar polarity patterning Development 2003 130 3007 3014 10.1242/dev.00526 12756182

[B40] Torban E Kor C Gros P *Van Gogh-like2 (Strabismus) *and its role in planar cell polarity and convergent extension in vertebrates Trends Genet 2004 20 570 577 10.1016/j.tig.2004.09.003 15475117

[B41] Kumburegama S Evolution of germ layers: insight from Wnt signaling in a cnidarian, *Nematostella vectensis* Ph.D. thesis 2009 University of Hawaii at Manoa, Zoology Department

[B42] Houliston E Momose T Manuel M *Clytia hemisphaerica*: a jellyfish cousin joins the laboratory Trends Genet 2010 26 159 167 10.1016/j.tig.2010.01.008 20227783

[B43] Momose T Derelle R Houliston E A maternally localized Wnt ligand required for axial patterning in the cnidarian *Clytia hemisphaerica* Development 2008 135 2105 2113 10.1242/dev.021543 18480163

[B44] Bisgrove B Yost HJ The roles of cilia in developmental disorders and disease Development 2006 133 4131 4143 10.1242/dev.02595 17021045

[B45] Gerdes JM Davis EE Katsanis N The vertebrate primary cilium in development, homeostasis, and disease Cell 2009 137 32 45 10.1016/j.cell.2009.03.023 19345185 PMC3016012

[B46] Koji K Yohei N Katsumi K Akira K Dishevelled, a Wnt signaling component, is involved in mitotic progression in cooperation with Plk 1 EMBO Journal 2010 29 3470 3483 10.1038/emboj.2010.221 20823832 PMC2964169

[B47] Frick JE Ruppert EE Primordial germ cells of *Synaptula hydriformis *(Holothuroidea; Echinodermata) are epithelial flagellated-collar cells: their apical-basal polarity becomes primary egg polarity Biol Bull 1996 191 168 177 10.2307/1542920 29220235

[B48] Frick JE Ruppert EE Primordial germ cells and oocytes of *Branchiostoma virginiae *(Cephalochordata, Acrania) are flagellated epithelial cells: relationship between epithelial and primary egg polarity Zygote 1997 5 139 151 10.1017/S0967199400003816 9276511

[B49] Maddison WP Maddison DR MacClade: Analysis of Phylogeny and Character Evolution 1992 Sunderland Mass: Sinauer 10.1159/000156416 2606395

[B50] Ronquist F Huelsenbeck JP MrBayes 3: Bayesian phylogenetic inference under mixed models Bioinformatics 2003 19 1572 1574 10.1093/bioinformatics/btg180 12912839

